# Novel effective and repeatedly available ring-thread counter traction for safer colorectal endoscopic submucosal dissection

**DOI:** 10.1007/s00464-016-5326-7

**Published:** 2016-11-17

**Authors:** Hirohito Mori, Hideki Kobara, Noriko Nishiyama, Shintaro Fujihara, Tae Matsunaga, Tsutomu Masaki

**Affiliations:** 1 Department of Gastroenterology and Neurology, Faculty of Medicine, Kagawa University, 1750-1 Ikenobe, Miki-cho, Kita-gun, Kagawa 761-0793 Japan; 2 Department of Gastroenterological Surgery, Ehime Rosai Hospital, Niihama, Ehime Japan

**Keywords:** Ring-shaped thread, Counter traction, Colorectal lateral spreading tumors, Endoscopic submucosal dissection

## Abstract

**Background:**

Although several methods to create an effective counter traction for safer endoscopic submucosal dissection (ESD) have been reported, these methods do not overcome problems regarding delivery and ease of use. This randomized prospective study assessed the usefulness of ring-shaped thread counter traction, which not only allowed the safer colorectal ESD but also the easiest and lower cost counter traction without any special devices.

**Methods:**

Forty-five patients diagnosed with colorectal lateral spreading tumors over 20 mm were allocated to the conventional ESD group (CE) (*n* = 22) and the ring-shaped thread counter traction ESD group (RE) (*n* = 21). The ring-shaped thread was hooked and lifted up to the contralateral mucosa with a hemoclip. The primary outcome was the dissected area per minute during ESD (cm^2^/min) (UMIN000020160).

**Results:**

There were significant differences in the dissection time (min), with 130.0 (56.0–240.0) versus 80 (35.0–130.0) min for the CE and RE groups, respectively (*P* = 0.001). For the dissected areas per minute (cm^2^/min), there was a significant difference, with 0.125 (0.1–0.18) versus 0.235 (0.16–0.36) min (*P* = 0.003) for the CE and RE groups, respectively. There were 1 cases of perforation during ESD in the CE compared to 0 for the RE, and this was no significantly different (*P* = 0.31). The procedure time of producing and setting the ring-shaped thread counter traction was approximately 1.80 (0.80–3.30) min only.

**Conclusions:**

The ring-shaped thread counter traction is simple, effective, lower cost and does not require special devices to obtain repeated counter traction.

**Electronic supplementary material:**

The online version of this article (doi:10.1007/s00464-016-5326-7) contains supplementary material, which is available to authorized users.

Endoscopic submucosal resection (ESD) is the standard treatment for large colorectal tumors in Japan. ESD allows *en bloc* resection of large lesions and provides accurate histopathological results for patients [1–3]. The perforation rate during ESD is around 2% [4, 5].

However, in European countries or the USA, evidence for the clinical value of ESD is limited and may not be directly applicable to Europe, where the results of ESD were reported less favorable due to the limited Western ESD expertise [6]. Moreover, perforation occurred in approximately 4.9% of patients for ESD and 0.9% for EMR, and bleeding occurred in 1.9% for ESD and 2.9% for EMR. Therefore, the overall need for further endoscopic recovery treatment and surgery because of complications (perforation and bleeding) was 7.8% for ESD and 3.0% for EMR [7]. Because the complication rate was very high, particularly for lesions with submucosal fibrosis, the perforation rate was reported as very high [8, 9]. Therefore, more effective and safer technical measurements of procedures or devices are needed to perform safer ESD and to disseminate this minimal invasive endoscopic technique worldwide.

As well as good counter traction, for post-ESD artificial ulcers, a prophylactic endoscopic post-ESD ulcer closure was significantly effective at preventing delayed perforation and providing good clinical courses for white blood cell count and levels of serum C-reactive protein after ESD [10–12].

In this prospective randomized study, we evaluated the usefulness of ring-shaped thread counter traction that allows endoscopists not only to perform colorectal ESD more safely under a clear operative view that may disseminate this minimal invasive endoscopic technique worldwide but also to provide the easiest and lower cost counter traction without the need of any special devices for all endoscopists.

## Patients and methods

Forty-five patients were diagnosed with colorectal tumors [lateral spreading tumors (LSTs)] over 20 mm in diameter by colonoscopy between December 2015 and July 2016 at either Ehime Rosai Hospital or Kagawa University Hospital and were enrolled after approval from the institutional review boards of each institution. Inclusion criteria were colorectal tumors with diameters over 20 mm in diameter [lateral spreading tumors (LSTs)] that were classified in the JSCCR (Japanese Society for Cancer of the Colon and Rectum) as follows: LST-G (granular type) and LST-NG (non-granular type). Exclusion criteria were definitely protruded-type tumors, such as (0–1), and a diagnosis of advanced colorectal (SM massive) cancer with image-enhanced magnified endoscopic examination. Tumor diameters were measured using measuring forceps. A total of two patients were excluded due to diagnoses of advanced colorectal (SM massive) cancer with image-enhanced magnified endoscopic examination. Finally, a total of 43 of 45 patients were included with colorectal tumors larger than 20 mm.

**Fig. 1:**
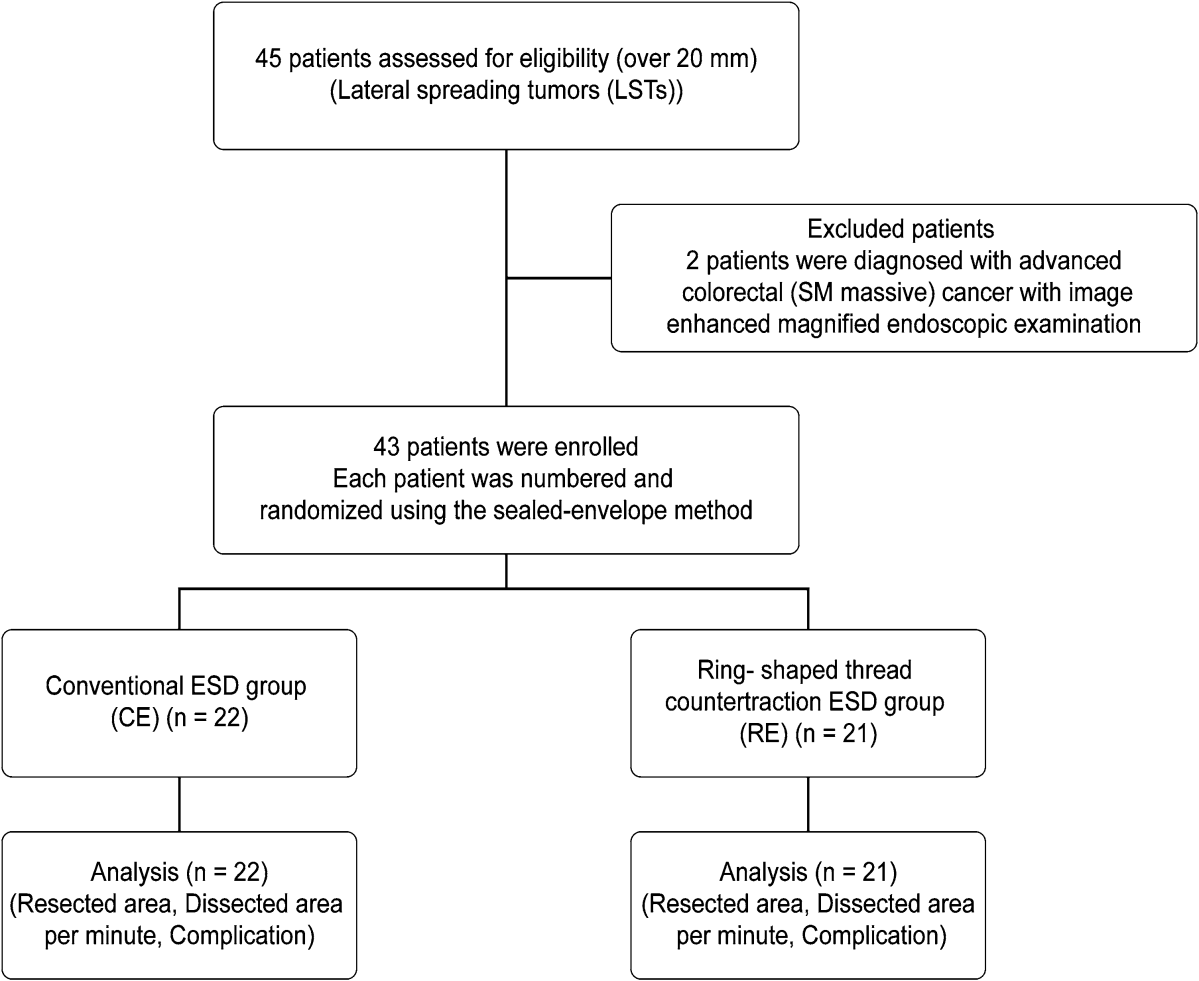
Allocation flowchart of enrolled patients. A total of 43 patients (43 lesions) were included out of 45 patients with colorectal LSTs larger than 20 mm. The lesions were randomly allocated to the conventional ESD group (CE) (22 lesions) or the ring-shaped thread counter traction ESD group (RE) (21 lesions) using the sealed-envelope method

The patients were randomly assigned numbers using the sealed-envelope method. Odd-numbered patients (*n* = 22) were allocated to the conventional ESD group (CE), and even-numbered patients (*n* = 21) were allocated to the ring-shaped thread counter traction ESD group (RE) (Fig. 1).

Patients who were taking anticoagulants were changed to heparin 4 days before ESD to maintain a prothrombin time–international normalized ratio (PT-INR) of 1.5, and heparin was discontinued 3 h before ESD. Heparin was resumed 3 h after ESD, and anticoagulants resumed the following day. Patients taking antiplatelet drugs consulted a cardiologist. Patients taking ticlopidine hydrochloride, clopidogrel sulfate or aspirin were changed to cilostazol 3 days before ESD, and they discontinued cilostazol the day of ESD. All antiplatelet drugs were resumed the day after ESD.

Colon pretreatment consisted of ingestion of 2 L of a polyethylene glycol solution (Niflec, Ajinomoto Pharma Co., Tokyo, Japan). All patients were discharged 6 days after ESD.

### Procedures of ring-shaped thread counter traction technique

**Fig. 2:**
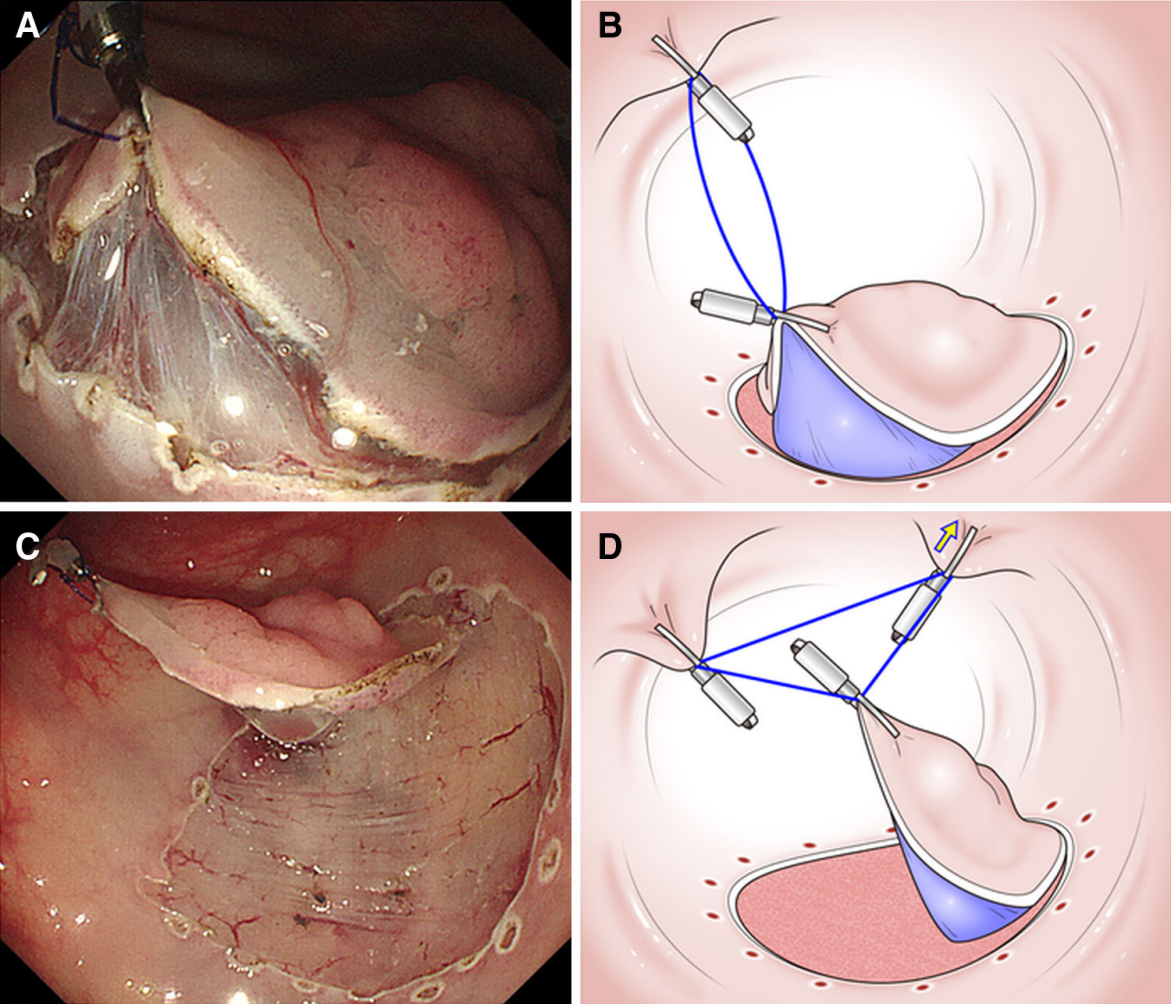
The typical picture and schema of ring-shaped thread counter traction. **A** By lifting up the edge of the lesion, it became easier and safer to begin the incision into the submucosal layer. **B** The schema revealed 10-mm ring thread as inserted into the colon through a channel for clipping both the affected sides of the colon, thereby lifting the lesion. **C** As submucosal dissection was continued and the traction force of the ring-shaped thread was decreased, a third hemoclip was added to hook and slide one side of the ring-shaped thread to obtain further counter traction. **D** As this schema reveals, additional counter tractions were repeatedly possible by adding hemoclips hooking and sliding contralateral to the lesion

**Fig. 3:**
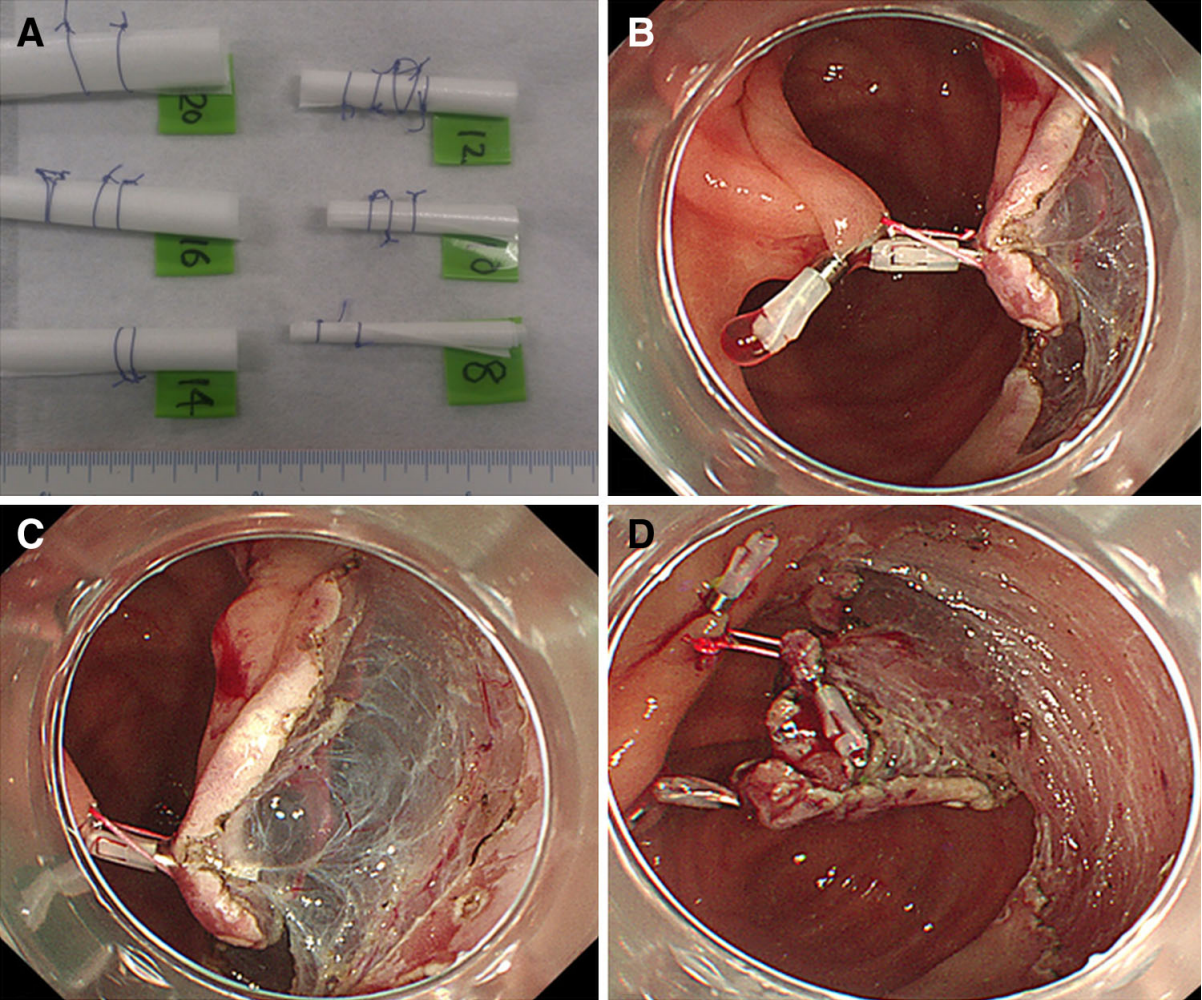
A typical case of ring-shaped thread counter traction in cecal LST-NG. **A** Various sized ring-shaped threads (8–20 mm) were prepared before ESD. **B** The ring-shaped thread was hooked and lifted up to the contralateral mucosa with a hemoclip by deflating air. **C** In proportion to the amount of insufflation of CO_2_, adjusting the strength of counter traction with the ring-shaped thread was possible. **D** As submucosal dissection was continued and the traction force of the ring-shaped thread was decreased, a second ring-shaped thread was placed to obtain additional counter traction force

The random allocation of patients to each group was conducted using sealed numbered envelopes prepared previously. The three endoscopists who performed ESD were members of the Japan Gastroenterological Endoscopy Society. All investigators received a lecture on the ring-shaped thread counter traction ESD methods. In the CE group, ESD was performed in the usual manner. Figure 2 shows a picture and schema of the ring-shaped thread counter traction. By lifting up the edge of the lesion, it became easier and safer to begin the incision into the submucosal layer (Fig. 2A, B). As submucosal dissection was continued and the traction force of the ring-shaped thread was decreased, a third hemoclip was added to hook and slide one side of the ring-shaped thread to obtain further counter traction (Fig. 2C, D). In the RE group, various sized ring-shaped threads (8–20 mm) were prepared (Fig. 3A). After a circumference mucosal incision was performed, 8-mm ring-shaped thread was placed through the endoscopic channel. The ring-shaped thread was hooked and lifted up to the contralateral mucosa with a hemoclip by deflating air (Fig. 3B). In proportion to the amount of insufflation of CO_2_, adjusting the strength of counter traction with the ring-shaped thread was possible (Fig. 3C). A second ring-shaped thread was placed if more counter traction was needed (Fig. 3D) (Video).

In both groups, the dissection time (DT*n*) (min) was defined as the dissecting time of submucosal layer only, except for the other ESD procedure times, which were measured by a nurse with a stopwatch who measured only the dissecting time when the ESD operator used electric knives during the ESD.

The shorter axis (abbreviated S*n*) (cm) and longer axis (abbreviated L*n*) of the ellipsoid resected specimen were measured after ESD. The ellipsoid resected area (abbreviated A*n*) was defined as the area calculated by the following formula:

$${\text{A}}n\left( {{\text{cm}}^{ 2} } \right) = \pi \times {\text{S}}n/2 \times {\text{L}}n/2\left( {\pi = 3.14} \right)(n = 1 - 43)$$

$${\text{The}}\,{\text{dissected}}\,{\text{area}}\,{\text{per}}\,{\text{minute}}\,{\text{during}}\,{\text{ESD}}\left( {{\text{DA}}n} \right)\,\left( {{\text{cm}}^{ 2} /{ \hbox{min} }} \right) = {\text{A}}n/{\text{DT}}n\,(n = 1 - 43)$$

### Ethical considerations

The Ethics Committees of Ehime Rosai Hospital and Kagawa University approved this study (approval no. 67) according to the Declaration of Helsinki. The patients provided verbal and written informed consent.

### Trial registration

University Hospital Medical Information Network (UMIN000020160) following the CONSORT check list.

### Study sample size and enrollment

After we conducted the pilot study for 8 patients in the CE and 8 patients in the RE (total 16 patients), we found significant differences between the two groups in the dissection speed (cm^2^/min). Based on the results of pilot studies (DAn) (cm^2^/min), the sample size was calculated by performing a statistical analysis using GraphPad Prism with the sample size (23 patients) using the effective size of 0.80 (standard deviation 0.0351) (*α* error 0.05, *β* error 0.20, power 0.8). (http://biostat.mc.vanderbilt.edu/wiki/Main/PowerSampleSize).

### ESD devices

- Endoscope: CF-HQ290 (Olympus Co., Tokyo, Japan).
- Incisional knife: IT knife 2 (KD-611L, Olympus Co., Tokyo, Japan).
- Hemostatic forceps: Coagrasper (FD-410LR, Olympus Co., Tokyo, Japan).
- Injection needle: NM-4U (Olympus Co., Tokyo, Japan).
- Local injection solution: hyaluronic acid (MucoUp^®^, Johnson & Johnson K.K., Tokyo, Japan) and 10% glycerin (mixing ratio 1:1).
- Incisional generator device: ERBE VIO300D (Elektromedizin, Tübingen, Germany).

### Outcomes

The primary outcome was the dissected area per minute during ESD (DA*n*) (cm^2^/min) (*n* = 1–43).

The secondary outcomes were as follows:

1. The incidence rate of post-ESD bleeding 1–7 days after ESD.
2. The incidence rate of perforation during ESD.
3. The setting and procedure time of ring-shaped thread counter traction during ESD.

### Statistical analysis

Data between groups were analyzed using Fisher’s exact test or the *χ* ^2^ test to compare the relative frequencies. The *t* test and Mann–Whitney *U* test were used to compare continuous variables with a significance level of *P* < 0.05. Statistical analyses were performed using GraphPad Prism version 5.00 for Windows (GraphPad software, San Diego, CA, USA).

## Results

Of the characteristic backgrounds in the CE and RE groups, there were no significant differences in age and gender (*P* = 0.23 and 0.48). For the location of lesions, in the CE group (22 lesions), we found 3 lesions in the cecum, 13 in the colon and 6 in the rectum. In the RE groups (21 lesions), 5 lesions were in the cecum, 9 in the colon and 7 in the rectum. There was no significant difference in the location of lesions (*P* = 0.15).

Macroscopic findings of lesions (JSCCR classification) revealed that LST-G (granular type) accounted for 12 and 14 lesions, and LST-NG (non-granular type) for 10 and 7 lesions in the CE and RE, respectively. There was no significant difference in the macroscopic findings (*P* = 0.13) (Table 1).

**Table 1:** Baseline characteristics

	Conventional ESD group (CE) (*n* = 22)	Ring-shaped thread counter traction ESD group (RE) (*n* = 21)	*P* value*
Age, years (mean ± SD)	72 ± 12	74 ± 10	0.23*
Gender (male/female)	14/8	16/5	0.48**
Locations of lesions			0.15**
Cecum	3	5	
Colon	13	9	
Rectum	6	7	
Macroscopic findings of lesions (JSCCR classification)			0.13***
LST-G	12	14	
LST-NG	10	7	

*LST* Lateral spreading tumor, *G* granular type, *NG* non-granular type, *JSCCR* Japanese society for cancer of the colon and rectum

* Unpaired *t* test, ** *χ* ^2^ test, *** Fisher’s exact test

**Fig. 4:**
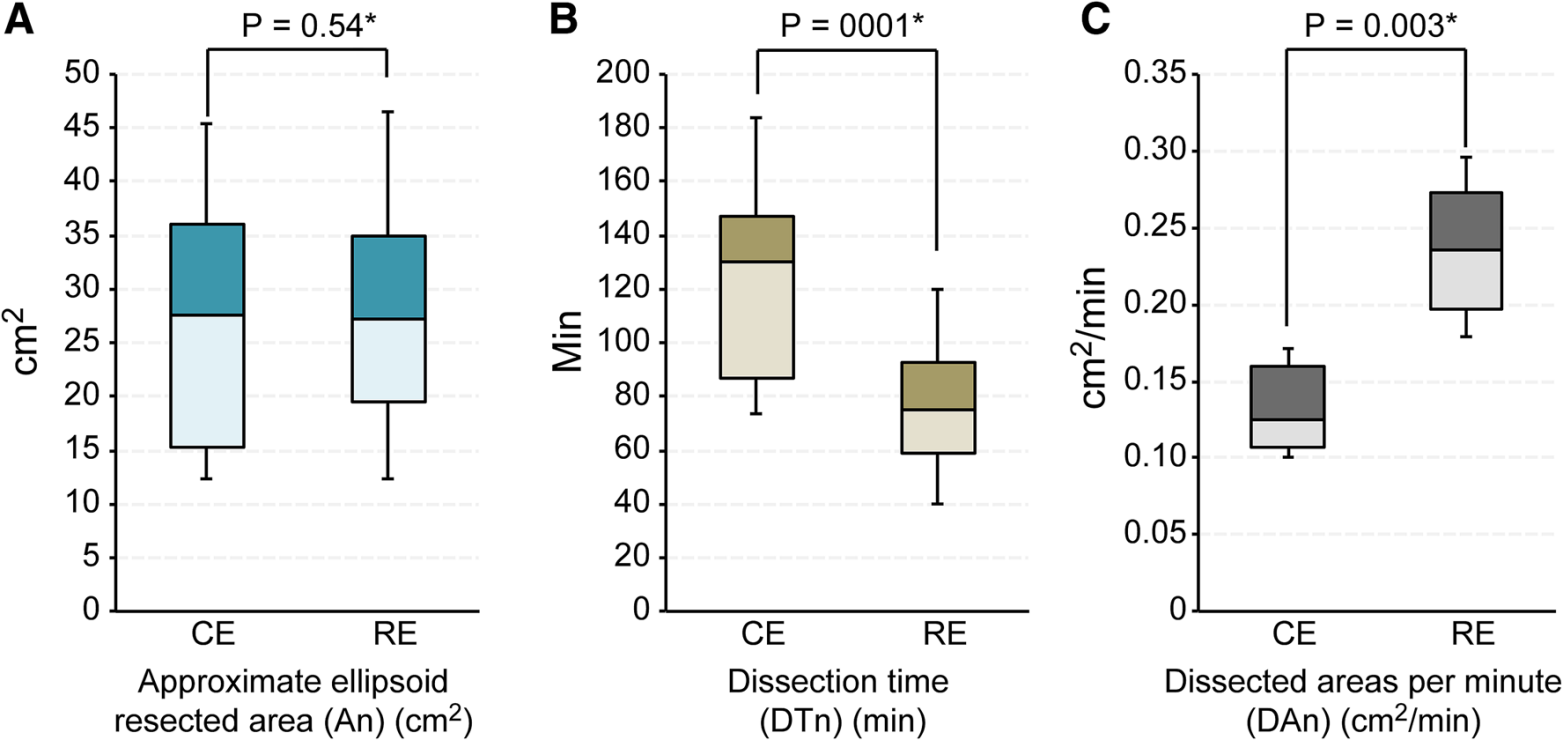
Diagrams of the two groups for resected area, dissection time and dissected areas per minute. **A** No significant difference was observed in the approximate ellipsoid resected area (A*n*) (cm^2^) between the CE and RE groups. **B** A significant difference in the dissection time (DT*n*) (min) was observed between the CE and RE groups. **C** In the dissected areas per minute (DA*n*) (cm^2^/min), there was a significant difference, with 0.125 (0.1–0.18) versus 0.235 (0.16–0.36) (cm^2^/min)

There were no significant differences in the approximate ellipsoid resected area (A*n*) (cm^2^), with 27.6 (10.3–50.20) versus 27.3 (11.0–49.9) cm^2^ for the CE and RE groups, respectively (*P* = 0.54) (Table 2) (Fig. 4A). There were significant differences in the dissection time (DT*n*) (min): 130.0 (56.0–240.0) versus 80 (35.0–130.0) (min) for the CE and RE groups, respectively (*P* = 0.001) (Fig. 4B). In the dissected areas per minute (DA*n*) (cm^2^/min), there was a significant difference, with 0.125 (0.1–0.18) versus 0.235 (0.16–0.36) (cm^2^/min) (*P* = 0.003) (Fig. 4C) for the CE and RE groups, respectively. There was 1 case of post-ESD bleeding (1–7 days after ESD) in the CE group compared to 0 in the RE group, which was of no significant difference (*P* = 0.31). There was 1 case of perforation during ESD in the CE groups compared to 0 in the RE group, which was also of no significant difference (*P* = 0.31) (Table 2). Perforation sites were successfully closed with hemoclips by ESD experts immediately without an emergency operation.

**Table 2:** Results according to with or without counter traction

	Conventional ESD group (CE) (*n* = 22)	Ring-shaped thread counter traction ESD group (RE) (*n* = 21)	*P* value*
The approximate ellipsoid resected area (A*n*) (cm^2^), median (range)	27.6 (10.3–50.20)	27.3 (11.0–49.9)	0.54*
Dissection time (DT*n*) (min), median (range)	130.0 (56.0–240.0)	80 (35.0–130.0)	0.001*
The dissected area per minute (DA*n*) (cm^2^/min), median (range)	0.125 (0.1–0.18)	0.235 (0.16–0.36)	0.003*
Post-ESD bleeding (1–7 days after ESD) (cases)	1	0	0.31*
Perforation during ESD (cases)	1	0	0.31*
The setting and procedure time of ring-shaped thread counter traction (min), median (range)	–	1.80 (0.80–3.30)	–
Histological categorization of polyps			0.42**
Adenocarcinoma (pM~SM1)	9	11	
Adenoma (high-grade atypia)	5	5	
Adenoma (low-grade atypia)	8	5	

* Mann–Whitney *U* test, ** Fisher’s exact test

The procedure time of making and setting ring-shaped thread counter traction to the lesions was approximately only 1.80 (0.80–3.30) min. Histopathological examinations revealed no significant differences in the proportions of adenocarcinoma, adenomas with high-grade atypia and adenomas with low-grade atypia (*P* = 0.42) (Table 2). There was no bleeding and any other complication at the opposite side of the lesion at the location of clipping.

## Discussion

In 2002, when the name of “ESD” had not yet been established, Oyama T. published the clip with line method, for the first time, and published several counter traction methods [13]. Since then, several similar methods to create an effective counter traction for a good operation view have been reported. Okamoto et al. [14] reported the cross-counter technique, which used the over tube equipped with an outer channel with the clip with line, was a useful method to introduce safer ESD without an expert of gastric ESD. Xie et al. [15] reported a similar method where the dissection time in 100 cases of esophageal ESDs was shorter in the clip traction group than in the non-clip traction group, and the rate of muscularis propria injury was reduced in the clip traction group. Using the modified clip with line methods, Noda et al. reported the thread traction with a sheath of polypectomy snare (TTSPS) reduced interference between the movement of the endoscope and the clip with line. This TTSPS made it possible to pull the lesion toward the anal as well as the oral side in gastric cancers [16, 17], and Yamasaki et al. [18, 19] and Yamada et al. [20] reported methods for colorectal cancers. As a randomized prospective study, Koike et al. reported that the thread-traction method was safe and shortened the dissection time and concluded that the thread-traction method was a safer and more useful procedure for esophageal cancers [21–23]. The main conclusions of these studies were that creating good working space for ESD between muscular propria and resected lesion reduced adverse event such as perforation and bleeding. As these methods enabled for endoscopist to perform ESD safer, faster and more accurate using clip with line thread method, over-tube method and snare method, these devices along with endoscope interfered with each other more or less, and easier to deliver and use method was needed. Therefore, several methods to create sufficient working space within the digestive tract were reported using tiny devices. Matsuzaki et al. [24] reported that the magnetic anchor-guided gastric endoscopic submucosal dissection (MAG-ESD) that were used in in vivo dog experiments created excellent counter traction for good visualization in the dog stomach. However, this system is not yet available for humans because the “magnetic anchor” is itself a foreign body in the human digestive tract. Ritsuno H et al. [25] reported the “S-O clip,” the advantages of which were good counter traction under the direct visualization of the cutting line and usage at any location without withdrawing the endoscope for colorectal cancers. This tiny device is equipped with a 5-mm spring attached between two clips and can be placed through the channel. It obtained good counter traction and worked independent of the interfering movement of the endoscope and thread, and its efficacy was confirmed in a prospective clinical trial. Because the concept of this S-O clip was similar to our ring-shaped thread counter traction, the crucial difference between these two methods is the concept of creating counter traction using insufflation expansion radial force in ring-shaped thread counter traction in contrast to the pulling force of a mechanical spring or rubber in the S-O clip [26]. Moreover, the cost and easy to use were important factors to spread these methods widely. For medical costs, the ring-shaped thread counter traction requires almost no cost other than thread and creating good counter traction oneself. Furthermore, it requires only 1.8 min on average to make the ring thread and place it into the lesion. This is a very simple method. Moreover, ring-shaped thread counter traction makes it possible to obtain counter traction force repeatedly by hooking the thread and clipping again and again, similar to a cat’s cradle, consistent with a decrease of counter traction force.

In conclusion, ring-shaped thread counter traction may achieve a lower cost of one’s own making without special devices, such as over tube or snare, to obtain repeated counter traction if needed.

## Electronic supplementary material

Below is the link to the electronic supplementary material.

Supplementary material 1 (MPG 51750 kb)
